# Algal Cell Factories: Approaches, Applications, and Potentials

**DOI:** 10.3390/md14120225

**Published:** 2016-12-13

**Authors:** Weiqi Fu, Amphun Chaiboonchoe, Basel Khraiwesh, David R. Nelson, Dina Al-Khairy, Alexandra Mystikou, Amnah Alzahmi, Kourosh Salehi-Ashtiani

**Affiliations:** 1 Division of Science and Math, New York University Abu Dhabi, P.O. Box 129188 Saadiyat Island, Abu Dhabi, UAE, wf21@nyu.edu (W.F.); ac4793@nyu.edu (A.C.); dak9@nyu.edu (D.A.-K.); amystikou@gmail.com (A.M.); 2 Center for Genomics and Systems Biology (CGSB), New York University Abu Dhabi, P.O. Box 129188 Saadiyat Island, Abu Dhabi, UAE, bhk1@nyu.edu (B.K.); drn2@nyu.edu (D.R.N.); amnah.alzahmi@nyu.edu (A.A.)

**Keywords:** algae, bioactive compound, cell factory, genetic engineering, mutagenesis, systems biology

## Abstract

With the advent of modern biotechnology, microorganisms from diverse lineages have been used to produce bio-based feedstocks and bioactive compounds. Many of these compounds are currently commodities of interest, in a variety of markets and their utility warrants investigation into improving their production through strain development. In this review, we address the issue of strain improvement in a group of organisms with strong potential to be productive “cell factories”: the photosynthetic microalgae. Microalgae are a diverse group of phytoplankton, involving polyphyletic lineage such as green algae and diatoms that are commonly used in the industry. The photosynthetic microalgae have been under intense investigation recently for their ability to produce commercial compounds using only light, CO_2_, and basic nutrients. However, their strain improvement is still a relatively recent area of work that is under development. Importantly, it is only through appropriate engineering methods that we may see the full biotechnological potential of microalgae come to fruition. Thus, in this review, we address past and present endeavors towards the aim of creating productive algal cell factories and describe possible advantageous future directions for the field.

## 1. Introduction

Microalgae have drawn great attention as a promising source for sustainable production of fatty acids, carotenoids, vitamins, and other compounds of interest [1]. Altogether, secondary metabolites from microalgae have great potential for industrial development as they include bioactive compounds such as antioxidant, antiviral, antibacterial, antifungal, anti-inflammatory, antitumor, and antimalarial effectors [2]. For example, nutritional lipids including essential fatty acids such as docosahexaenoic acid (DHA) have been commercially produced from microalgae [3]. However, natural products in microalgae remain largely unexplored compared to those in land plants [2], even though cultivation of microalgae offers many advantages over those of terrestrial plants, e.g., the rapid growth rates, and lack of competition for resources used for food crops, including the use of fresh water and arable lands. 

The important fact that microalgae have been granted the GRAS (Generally recognized as safe) status opens the path wide for the use of microalgae as an attractive cell factory. The U.S. FDA (Food Drug Administration) issues a GRAS certificate and grants this “safe to consume” status depending on studies and scientific literature and evidence that the material in question is not harmful under predetermined conditions of use. This GRAS classification is critical as it cut costs of downstream purification of compounds or proteins purified as this status eliminates the need for further purification steps. As such, many algae species are considered GRAS [4]. For instance, *Spirulina* has been recognized as GRAS by the FDA with no risk for human health since 1981 [5]. Species of green microalgae such as *Chlorella* and *Dunaliella*, which are a rich source of vitamins, lipids, and other bioactive compounds, are also considered GRAS by FDA [5].

**Figure 1:**
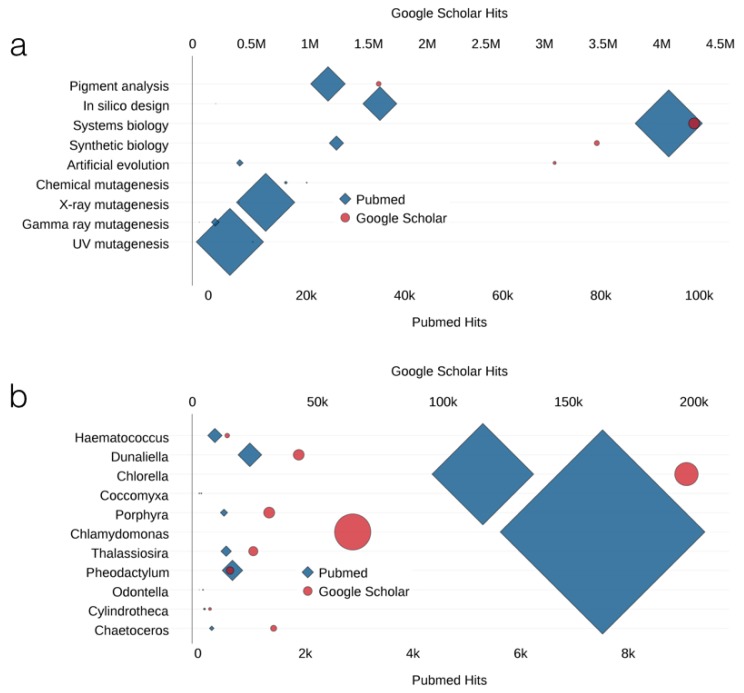
Trends in algal research: (**a**) Results of queries in PubMed and Google Scholar search engines with topic terms are shown on *x*-axes (bottom, PubMed; top, Google Scholar); the number of hits for topic term “AND algae” are indicated by size. (**b**) The number of hits for algal species are shown on the *x*-axes (bottom, PubMed; top, Google Scholar); the number of hits for algal species AND (synthetic OR systems OR *in silico* OR artificial evolution OR mutagenesis OR pigment) are indicated by size.

**Figure 2:**
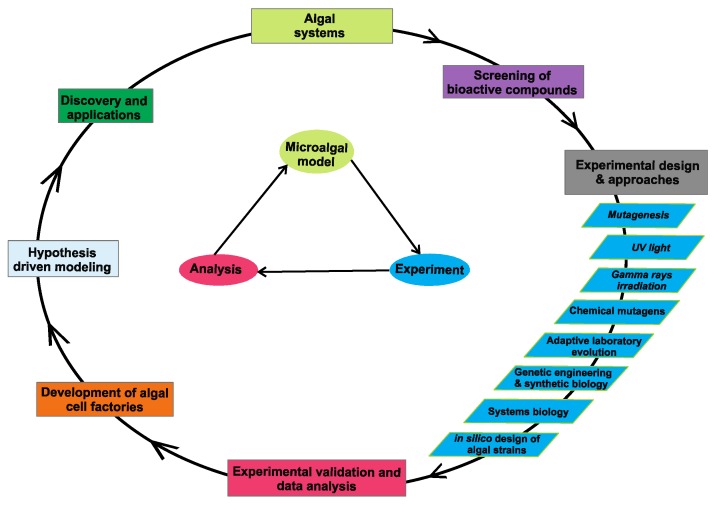
A conceptual representation of an integrative strain-engineering approach. Various experimental and -omics datasets are integrated with metabolic modeling for the development of algal cell factories.

The microalgae have proven to be of considerable interest as a source of bioactive compounds and applied research in this field is growing dramatically (Figure 1). Although genetically unmodified microalgae may be exploited for the production of particular metabolites that they can accumulate, it is expected that strain development is required to make industrial production feasible. Therefore, there is a need to develop new production strains with features such as fast growth, high tolerance to lights and heat, etc. To date, some approaches have been developed to increase algal productivity for value-added bioproducts. Mutagenesis, adaptive laboratory evolution (or ALE), and genetic engineering have been established as strategies for developing algal cell factories. Furthermore, systems biology and synthetic biology perspectives and approaches are emerging in the production of bioactive compounds, such as carotenoids and vitamins, in algae [6,7]. In addition, using *in silico* metabolic models to complement and contextualize genetic and metabolic data will provide researchers more options for the strain improvement of target products [8,9,10,11]. However, the progress in the field is not well documented, and significant achievements on developing algal cell factories are yet to be recognized. In this article, we review, and comment on recent progress on metabolic engineering and strain improvement in microalgae research with examples using mutagenesis, adaptive evolution, genetic engineering, and systems biology approaches (Figure 2). 

## 2. Approaches for Developing Algal Cell Factories

Microalgae are photosynthetic eukaryotic organisms, which may be drawn up as model organisms for the sustainable production of fine chemicals such as nutritional supplements, carotenoids and polyunsaturated fatty acids (PUFAs) [12]. There are many parameters that can be used to increase pigment and lipid production in microalgae, for example, nutrient starvation, light stress and other stress conditions. In this section, current and different types of physical and chemical mutagens to develop algal cell factories will be reviewed with specific examples. Further, emerging technologies in the field for strain development will be discussed.

### 2.1. Mutagenesis

Mutagenesis in laboratories is a process in which a physical or chemical mutagen is used to induce a higher frequency of mutation than the natural rate of a particular organism. Then, stable mutants with improved traits may be screened and selected with changed and inheritable genetic information thereafter for the development of desired new variants [13]. Many physical mutagens including UV light and gamma and X-rays, as well as chemical mutagens, have been successfully applied on microalgae for improving strain performance [13].

#### 2.1.1. UV Light

Ultraviolet [14] light induces specific mutations that are usually caused by the induced formation of pyrimidine dimers on the same strand of DNA [15]. After exposure to either UVA or UVB, mutation is observed occurring preferentially at methyl-CpG sites [15]. UV mutagenesis is still a useful method in microalgae mutagenesis since it can be employed without clear genetic information from microalgae species. Compared with chemical mutagenesis methods, UV mutagenesis can be controlled more flexibly to avoid secondary contamination. Previous studies have shown that UV mutagen on organisms achieved *Dunaliella bardawil* mutant strains that are rich in β-carotene. UV mutagenesis also increased the eicosapentaenoic acid [16] accumulation in the diatom *Phaeodactylum tricornutum* [16] and EPA and docosahexaenoic acid production [17] in *Pavlova lutheri* [18], respectively. UV mutagenesis has also been shown as an efficient method to improve both the biomass and the lipid content in *Chlorella* strains [18].

We recently developed and demonstrated the application of UV mutagenesis integrated with fluorescence-activated cell sorting (FACS) for selection, and confocal Raman microscopy for lipid analysis in *Chlamydomonas* as a model for generating lipid-accumulating microalgae [19,20]. Raman microscopy enabled quantitative determination of the unsaturation levels and chain lengths of microalgal lipids, which are vital parameters in selection and engineering of microalgae for optimal production of biofuels. The obtained results demonstrated the presence of stable clonal differences on saturation status of expressed lipids [19,20]. 

#### 2.1.2. Gamma Rays Irradiation

Gamma rays are very short wavelengths obtained by the disintegration of the radioisotopes ^60^Co, ^123^I or ^137^Cs. Most gamma sources are suitable for seed irradiation, as long as the size of irradiation space is sufficient and the dose rate allows reasonable irradiation times [13]. For instance, breeding via gamma irradiation has been used to develop a high lipid-producing *Scenedesmus dimorphic* mutant [21].

#### 2.1.3. Chemical Mutagens

Chemical mutagens induced mutagenesis may be particularly biased in some cases, as they may increase the mutation levels, particularly in some genomic regions with high GC-content. It is expected that some mutagenesis experiments may not result in desired phenotypes due to limited mutant pools. Therefore, it is important to understand the biases before selection of a chemical mutagen for mutagenesis experiments [22].

*N*′-nitro-*N*-nitrosoguanidine (NTG) is a common chemical mutagen that has been widely used in *Escherichia coli* and other bacteria such as *Corynebacterium glutamicum* [22]. NTG has also been successfully used to generate mutants with enhanced carotenoid accumulation and increased astaxanthin content in the green alga *Haematococcus pluvialis* [23]. Ethyl methanesulfonate (EMS) is another popular chemical mutagen that has been proven to be effective and efficient in mutagenesis [13]. Successful mutagenesis has been performed using EMS to create algal mutants with increased lipid accumulation. For example, *Chlamydomonas reinhardtii* was randomly mutagenized by EMS for enhanced lipid production [24].

### 2.2. Adaptive Laboratory Evolution

Adaptive laboratory evolution (ALE) has been widely utilized for developing novel biological and phenotypic functions and also for strain improvement in synthetic biology for prokaryotic microorganisms that have mostly consisted of bacteria strains [25,26,27]. ALE experiments under well-controlled laboratory conditions followed by genome re-sequencing allowed us to study the genetic basis underlying adaptation to environmental stress. With the aid of cost-effective genome re-sequencing, it becomes feasible to identify the mutations occurred under selection pressure during ALE.

ALE approaches have also been applied to adaptively evolve eukaryotic microalgae to grow under controlled light conditions for increased carotenoid accumulation [28,29]. Numerous studies have been reported on the adaptation of cyanobacteria to abiotic stressors such as long-term thermal tolerance, butanol treatment, acid stress and high light stress [30,31,32]. However, these studies have mainly focused on identification of genetic modifications leading to the new phenotypic strains and rarely put any efforts on developing strains for improved production of bioactive compounds in cyanobacteria. 

In green microalgae, the nuclear, chloroplast and mitochondrial genomes of the model species *C. reinhardtii* have been fully sequenced and annotated. Adaptive evolution studies on *C. reinhardtii* strains were dedicated to lipid metabolism for high lipid accumulation or biofuel production [33,34,35]. Using a long time iterative light stress or ALE yielded *D. salina* strains with increased accumulation of carotenoids including β-carotene and lutein [28]. It has also been reported that ALE improved the high CO_2_ tolerance of *Chlorella* sp. and achieved increased accumulation of chlorophylls and carotenoids [36]. 

Diatoms have a different evolutionary history compared to green microalgae. They are thought to have evolved from an ancient secondary endosymbiosis between heterotrophic and autotrophic eukaryotes [37,38]. Diatoms containing different pigments different from green algae and higher plants in the light-harvesting complex are capable of generating significant amounts of specific compounds in response to fluctuations in environments, particularly about changes in illumination. Blue light is believed to be essential for high light acclimation in *P. tricornutum* [39], but the molecular basis of responses to light in marine diatoms is still largely unknown [16]. It is thought that high light acclimation in *P. tricornutum* is triggered by the redox state of the plastoquinone pool, similar to green algae and land plants [40]. A recent study showed that *P. tricornutum* strains developed by ALE achieved higher biomass production and enhanced fucoxanthin accumulation under combined red and blue light conditions, but the genetic mutations which may be potentially responsible for the altered phenotypes have not been deciphered yet [29].

In addition to the typical groups of microalgae, other marine species may be able to respond to environmental changes through adaptive evolution. For example, the coccolithophore *Emiliania huxleyi*, one marine alga that generates calcite scales biogenically and can produce alkenones, a compound associated with resistance to environmental stress, evolved in response to ocean acidification in 500 asexual generations and exhibited higher growth rates in adapted cultures, compared with the non-adapted culture [41]. 

In short, to conduct ALE on microalgae for strain optimization, one may need to select and determine useful selection parameters. These parameters involve growth-rate selection pressures such as light stress and nutrient depletion, solvents treatment such as using butanol as well as antibiotics, depending on the metabolic pathways that one may target. Intensive genome sequencing may help to reveal the genetic basis for adaptation of microalgae to environmental stress. For a comprehensive and in-depth understanding and application of ALE, genetic engineering and synthetic biology approaches may be developed to reintroduce point or combined mutations into wild-type starting strains for determining specific phenotypic consequences.

### 2.3. Genetic Engineering 

This section focuses on the progress and emerging technology of genetic engineering in eukaryotic microalgae. Eukaryotic microalgae possess all the advantages of photosynthetically driven systems but lack many of the disadvantages of plant-based expression systems, i.e., they have higher growth rates, are easy to grow and do not compete for lands with crops in agriculture [42]. The first and best-studied eukaryotic microalga is the soil and freshwater species *C. reinhardtii*, which its nuclear genetic manipulation system has been well established [43,44]. The enzyme β-carotene ketolase from *H. pluvialis* has been expressed in *C. reinhardtii* to synthesize the carotenoids ketolutein and adonixanthin [45]. In addition, the nuclear transformation of *C. reinhardtii* using genes isolated from *Chlorella zofingiensis* was developed, and engineering *C. reinhardtii* with a foreign phytoene synthase led to an increase of the carotenoids violaxanthin and lutein which were 2.0- and 2.2-fold higher than in untransformed cells [46,47]. 

Molecular biology tools for gene manipulation in diatoms have also been developed [42,48]. Microprojectile bombardment and electroporation have been applied successfully to introduce foreign DNA into *Phaeodactylum* cells for which the full genome sequence is available [49]. A shuttle vector (pPha-NR) with an inducible nitrate reductase promoter system (GenBank: JN180663) has been constructed for controllable expression of foreign genes [42]. Recently, the diatom *P. tricornutum* was engineered to accumulate the high value omega-3 polyunsaturated fatty acid docosahexaenoic acid [17] by incorporating the Δ5-elongase and acyl-CoA-dependent Δ6-desaturase genes from the picoalga *Ostreococcus tauri* [50]. 

Another well-established DNA delivery technique is transformation by *Agrobacterium tumefaciens*, a soil pathogenic bacteria that causes crown gall disease [51]. In recent years, this method has been successfully applied in algae [52]. Stable nuclear genetic transformation of *C. reinhardtii* mediated by agrobacterium has been reported [51]. Agrobacterium-mediated transformation has shown success in oil-bearing marine algae *Parachlorella Kesslerri* [52] and *Schizochytrium* [53], of which the latter is used for commercial production of oil rich in DHA. 

Using reverse genetics approaches such as homologous recombination, genetic engineering strategies using microalgae for enhanced production of value-added compounds can be developed. More precise genome editing tools such as zinc-finger nuclease (ZFN), transcription activator-like effectors (TALEs) and clustered regularly interspaced short palindromic repeats (CRISPR) have also been developed for gene activation, deletion and replacement in organisms as emerging technology. However, only a limited number of studies have been reported on engineering microalgae through these advanced genome editing tools, for example, ZFN-mediated gene editing in the green microalga *C. reinhardtii* and the use of TALEs in the marine diatom *P. tricornutum* [54]. It was reported that using CRISPR/Cas9 system for targeted gene modification in *C. reinhardtii* succeeded with an expression of Cas9 and single guide RNA (sgRNA) genes while the study suggested the failure to recover transformants of *C. reinhardtii* was caused by the toxicity of Cas9 produced constitutively following gene editing [55]. More recently, it was shown that delivering Cas9 ribonucleoproteins (RNPs) comprising the Cas9 protein and sgRNAs in contrast to vector-driven expression of Cas9 can improve the targeting efficiency of CRISPR/Cas9 in Chlamydomonas [56]. Another study reported the development of a CRISPR/Cas9 based system to create stable gene knockouts in the marine diatom *P. tricornutum* [57]. It appears much effort is needed to develop the genome editing technology that works effectively in microalgae.

Synthetic biology is an emerging research technology that enables us to redesign and construct biological devices and systems for producing target compounds [58]. However, synthetic biology is still a young field undergoing rapid development. Currently, due to the lack of highly efficient and effective tools for modification of target genes in the nuclear genome in microalgae, more investigations are required to fully exploit the potential of microalgae through various strategies [58,59]. Nevertheless, synthetic biology is very likely to play a major role in developing microalgae for bioactive compounds in the coming years. Recently, one of the diatom *P. tricornutum* chromosomes was successfully generated synthetically and maintained in yeast. The applications of these techniques will likely offer opportunities to reverse-engineer, then redesign a variety of algal species at genome-scales towards industrial applications [58]. 

### 2.4. Systems Biology and In Silico Design of Algal Strains

Systems biology is based on the large-scale high-throughput quantitative omics technologies including genomics, proteomics, metabolomics and transcriptomics and bioinformatics tools lead to a more comprehensive understanding of how the metabolisms varied in different environmental conditions, functional diversity and biosynthetic capacity [60]. Systems biology has become a valuable approach for understanding cellular metabolism, identify potential enzymes and pathways for metabolic engineering targets and improve microalgal strains to increase their productivity especially in the production of complex natural products [61,62].

Using next-generation sequence data, genome-scale metabolic model (GSM) for different organisms have been reconstructed and are being updated as new toolboxes are being developed [63]. The *in silico* genome-scale models have also enabled development of metabolic engineering strategies to optimize algal strains by improving and maximizing the yield of bioactive compounds of interest [64]. Pathway Tools [65] is one of the available software that can be used to manage, analyze, simulate and visualize the pathways, as well as for prediction of metabolic routes of the compound of interest. Constraint-based reconstruction and analysis (COBRA) [63] methods have become well known and widely used tools for metabolic and genetic engineering. GSM using COBRA to compute optimal flux distribution through the flux balance analysis (FBA) and strain optimization tools such as OptORF [66], EDGE [67] and Optknock [68] to predict gene overexpression and/or gene deletion, has been applied in identifying good strain candidates and generating hypothesis-driven strain engineering to achieve improved yield of target products. To date, some GSM have been reconstructed on algae but not yet fully utilized in bioactive compound production. Among microalgae, *C. reinhardtii’s* GSM is a widely used model, which has been reported, refined and updated some times [64,69,70]. Furthermore, the GSM for green picoalga *Ostreococcus* including two species *O. lucimarinus* and *O. tauri* were constructed [71]. In addition, GSM model of *P. tricornutum* has been developed [72] and recently updated to “*i*LB1027_lipid” model [73]; the model was utilized to understand the species response to light intensity [74]. 

*Chlorella* spp., a unicellular green microalga that is widely distributed in freshwater has been recognized as a promising candidate for biomass, biofuel, and value-added chemical production. *C. protothecoides* sequence available in 2014 [75] and primary metabolic model reconstruction is currently available (272 metabolic reactions, 270 enzymes, and 461 encoding genes and 190 metabolites) [76]. *C. variabilis*, *i*AJ526, was reconstructed with 526 genes, 1445 reactions and 1236 metabolites based on *C. variabilis* NC64A strain under three light sources [77]. The model for *C. vulgaris* UTEX 395, *i*CZ843, is the most comprehensive reconstruction (843 genes, 2294 reactions, and 1770 metabolites) [78] of the *Chlorella* models. Importantly, this reconstruction makes use of the Biolog phenotype microarray platform [70,79] to validate some reactions and pathways. 

*Arthrospira* is a genus of cyanobacteria that has seen a significant advance in its application in various fields including food, fuel and pharmaceutical industries. The iAK692 model is the first comprehensive GSM for *Spirulina (Arthrospira platensis)* that was derived from partial genome sequences through a semi-automated process based on the Pathway Tools software. The model contains 692 open reading frames (ORFs). This research provides an example of how the models can improve the industrial use of this strain [17]. Cyanobacteria are also a group of organisms with potential for development of cell factories. Their comprehensive genome-scale metabolic models have been published [80,81] and simulated to explore how carbon and energy are distributed. The iSyf715 model is a GSM model for *Synechococcus elongatus* PCC7942 [82], and iSyn669 [83] and iJN678 [84] are GSM for *Synechocystis* sp. PCC 6803. A novel gap-filling tool, MetabolIc Reconstruction via functionAl GEnomics (MIRAGE), has been applied to *Synechocystis* sp. PCC 6803 with a computational metabolic engineering tool called Optknock [68] to enhance astaxanthin production [85]. Recent web-based tools for system biology analysis for cyanobacteria can be found in recent reviews [86] and their application to the synthesis of industrial products was reported [87,88].

GSM of other organisms has been used to clarify DHA [17] biosynthesis mechanism and improve DHA production using iCY1170_DHA model of *Schizochytrium limacinum* SR21 [89]. A Gram-positive *Bacillus subtilis* iBsu1147 model also showed how the model could help to increase the following products: riboflavin, cellulase Egl-237, (*R*,*R*)-2,3-butanediol, and isobutanol [90]. GSM may also help to study the adaptation process since the optimal growth solutions can be computed from GSM and then utilized to interpret changes observed by comparing transcriptomic patterns from the wild-type and ALE strains.

Altogether, microalgae are promising sources of bioactive compounds, but there are still challenges to be addressed. With complete genome sequences of microalgae, available genetic manipulation tools, genome-scale metabolic models, omics data analyses can lead to a better understanding of biosynthetic pathways and optimization of the strains for enhanced production of target products.

## 3. Cell Factory Potentials in Macroalgae and Lower Plants 

In addition to microalgae, macroalgae and mosses are briefly discussed as potential photosynthetic cell factories in this section for their applications in biotechnology. Macroalgae constitute a renewable resource used by the food industry, as feed for animals, to produce phycocolloids, as cosmetic ingredients and for pharmaceutical applications. The moss *Physcomitrella patens*, which is a bryophyte, occupies a key evolutionary position bridging the gap between green algae and higher plants [91]. Because the protonema stage of the species grows quickly and simultaneously, it can be cultivated in a bioreactor as a genetically stable cell suspension. *Physcomitrella* can be easily manipulated using standard molecular biology methods and is haploid in a vegetative state, making it a highly suitable system for engineering strains towards industrial applications [92,93].

### 3.1. Macroalgal Species

A promising group of phototrophic organisms for biotechnology applications are the macroalgae (brown, Phaeophyta; red, Rhodophyta; and green, Chlorophyta) as they constitute diverse sources of natural products. Trosset and Carbonell [94] suggest and discuss the application of systems biology to red macroalgal species that have been identified as rich sources of diverse and novel bioactive compounds for drug development. Many of these molecules have high commercial value for nutritional supplements, specialty pigments, industrial polysaccharides and aquaculture [95]. Others produce bioactive compounds including ones with antibacterial, anti-tumor, antiviral and antifungal activities [96]. For example, many brown (e.g., *Laminaria saccharina*) and red (e.g., *Agardhiella subulala*) marine macroalgae produce bioactive compounds such as eicosanoids, which have substantial bioactivity against inflammation, asthma, heart diseases and cancer. A few other macroalgal species, such as members of *Ochtodes*, *Plocamium*, and *Portieria*, produce halogenated monoterpenes that may provide anti-tumor activities [97]. These macroalgal species are potential candidates for cell factory development. Thus far, stable and transient genetic transformations have been developed for seven macroalgal species to change or enhance their bioactivity. These species are: *Pyropia yezoensis* (Table 1), *Porphyra miniata*, *Kappaphycus alvarezii* and *Gracilaria changii* from the red algal phylum; *Saccharina japonica* (Table 1) and *Undaria pinnatifida* from the brown phylum; and the green alga *Ulva lactuca* [98]. The species above are prime candidates for future cell factory developments due to the availability of their genetic information; this is an advantage that can offer a better understanding for genetically engineering them. While the genomic knowledge about macroalgae is limited, the complete genomes of *Ectocarpus siliculosus*, *Pyropia yezoensis*, *Gracilariopsis lemaneiformis*, *Chondrus crispus* and *Saccharina japonica* have been sequenced; these can serve as model species to established genetic engineering in macroalgal species [99]. 

According to the Food and Agriculture Organization of the United Nations [100], algae constitute a large fraction of the global source for food production, pharmaceuticals, cosmetics and fertilizers, and are processed to extract thickening agents or used as an additive to animal feed with high commercial value. Algal farming is practiced for 37 different species in about 50 countries with a total estimated annual value of US$6.4 billion. Macroalgae such as *Pyropia* spp. have high nutritional value and are being used extensively for consumption especially in Asian countries. Farming is producing up to 27 million tons of macroalgae for commercial use, and it has been expanded by eight percent per year over the past decade. 

### 3.2. The Moss P. patens

Over the past 20 years, the moss *P. patens* has been developed as a model species in basic research and biotechnology [101,102] and can be a good candidate for the production of natural products, which are difficult of access. Several human proteins are being produced in this system as potential biopharmaceuticals [102]. Among the products are tumor-directed monoclonal antibodies with enhanced antibody-dependent cytotoxicity (ADCC), vascular endothelial growth factor (VEGF), complement factor H (FH), keratinocyte growth factor (FGF7/KGF), epidermal growth factor (EGF), hepatocyte growth factor (HGF), asialo-erythropoietin (asialo-EPO, AEPO), alpha-galactosidase (aGal) and beta glucocerebrosidase (GBA) [102]. The first moss-made pharmaceutical, aGal to treat Morbus Fabry, is in clinical trials [102]. Subsequently, a mutant was engineered to further “humanize” the moss glycosylation pattern by the expression of a human beta-1,4-galactosyltransferase gene. This gene was integrated into the *Physcomitrella patens* genome by “knockin” into the xylosyltransferase or fucosyltransferase locus [103]. To avoid unwanted *O*-glycosylation of human proteins produced in moss, a gene responsible for prolylhydroxylation was identified and deleted from the genome [104]. The first human protein produced in the moss system was the vascular endothelial growth factor (VEGF) [105], which has a central function in angiogenesis and cancer [106]. Several human growth factors (FGF7/KGF, EGF, and HGF) that are used in mammalian cell culture have been produced in the moss system [107]. We note that FGF7/KGF (keratinocyte growth factor) is the first commercially available moss-made human protein, intended for in vitro use. Based on these experiences, Moss has been suggested as a potential production host for vaccines [108], vaccine-producing moss may be directly administered as an oral vaccine. The first moss-made candidate vaccine is a chimeric Env-derived HIV multi-epitope protein that is immunogenic in mice [109]. An important issue in good manufacturing practice (GMP) is the molecular characterization of the producing cell factories. Once characterized and approved, subsequent production has to rely on identical clones that have to be stored in master cell banks. This can easily be achieved for clonal moss tissues, as they can be stored in liquid nitrogen and survive this cryopreservation to 100% even after many years [110]. 

## 4. Concluding Remarks and Perspectives

Microalgae have been recognized for their potential applications in industry. It is feasible to use microalgae in manufacturing facilities, converting CO_2_, water and sunlight to bioactive compounds such as carotenoids and fatty acids. However, from a critical point of view for sustainability, certain hurdles for using this particular production system needs to be addressed, for instance, the capacity for different product lines and the low photosynthetic efficiency and productivity. In tackling many of these challenges, systems biology and synthetic biology approaches may have great potential in developing photosynthetic cell factories effectively. The advancement of genetic engineering tools and the availability of algal genome data allow new algal species to be engineered and hence used as powerful cell factories for producing novel products of pharmaceutical and industrial value. Furthermore, the evolving omics technologies may lead to data-driven design and strain development on microalgae with reasonable efficiency. We anticipate that the rapid development of these innovative technologies will offer further opportunities for producing active pharmaceutical ingredients as well as discovering new therapeutic compounds in microalgae.

To summarize, microalgae have been regarded as promising and powerful cell factories for their significant role in global sustainability initiatives such as sustainable industry, sustainable agriculture, and ecological economics. Large-scale production using microalgae requires shorter periods of cultivation time in comparison with terrestrial plants, with many microalgae having doubling times of less than 24 h while land crops are seasonal. In addition, many algae have been granted the GRAS status, which makes the use of microalgae as cell factories for industrial and pharmaceutical purposes very attractive [150]. For engineering these microalgae, using forward genetic approaches may not affect their GRAS status. Even using genome-editing technology such as CRISPR/Cas9 system may fall outside the GMO legislation as long as no foreign DNA has been introduced into their genomes [151]. However, the GRAS status could be invalid if substances are expected to become components of the algal products as a result of genetic modification and the composition of such products has been changed [152]. Moreover, microalgae are still being considered as an alternative solution for the food versus fuel when used to produce biofuels instead of plant-derived biofuels that use fertile land [153]. Furthermore, microalgae can be grown in enclosed photobioreactors, which grants full containment of the genetically modified strains. In contrast, a significant level of concern remains on the use of transgenic plants because open land is used for their cultivation. These advantages and the high nutritional and pharmaceutical values that microalgae have, along with the broad range of antimicrobial bioactive compounds that algae contain, makes algae the best candidate for development of cell factories. 

## Figures and Tables

**Table 1:** Phototrophic species with available genome sequences or ongoing genome sequencing projects and additional information (where exist) about their environment, bioactive compounds, and properties/extracts. Data presented are available in the NCBI genome database (http://www.ncbi.nlm.nih.gov/) and the AlgaeBase website (http://www.algaebase.org/) [111].

Species	Group	Environment	Bioactive Compounds	Properties	Genetic Tools	Ref. ^1^
*Bigelowiella natans*	Cercozoa	marine			Genome; transcriptome under diurnal cycle; Viral elements known	[112,113,114]
*Chlorella* sp.	Chlorophyta	freshwater	Lipids, human growth hormone	Human nutrition, biofuels, medicines	Genes for active enzymes cloned; in silico models	[115,116,117,118,119]
*Coccomyxa subellipsoidea*	Chlorophyta	freshwater	Flounder growth hormone	Flounder fry exposed to Chlorella-expressed fGH for 30 days exhibited a 25% increase in both total length and width	Trans gene expression	[115,120]
*Ostreococcus lucimarinus*	Chlorophyta	marine	asymmetric carotenoids	Antioxidant molecules, human nutrition	Trans gene expression	[121,122]
*Micromonas pusilla*	Chlorophyta	marine			Trans gene expression, cDNA libraries	[123]
*Volvox carteri f. nagariensis*	Chlorophyta	freshwater			Trans gene expression, cDNA libraries	[124]
*Chlamydomonas reinhardtii*	Chlorophyta	freshwater		Human nutrition, biofuels	Trans gene expression, cDNA libraries	[125,126]
*Emiliania huxleyi*	Haptophyta	marine	Calcium carbonate, dimethyl sulfoxide	Human nutrition, weather influence	cDNA libraries	[127,128]
*Guillardia theta*	Cryptophyta	marine			Functional genes cloned for trans-expression in *E. coli*	[129]
*Nannochloropsis gaditana*	Chrysophyta	marine	lipids	Human nutrition, biofuels	Trans gene expression	[130,131]
*Ectocarpus siliculosus*	Phaeophyta	marine			Trans gene expression	[132]
*Saccharina japonica*	Phaeophyta	marine	porphyrin derivatives (pheophorbide a, pheophytin a)	anti-inflammatory activity	SNP linkage map	[2,133]
*Thalassiosira oceanica*	Phaeophyta	marine			cDNA libraries, RNAi, cloning of functional genes	[134]
*Thalassiosira pseudonana*	Phaeophyta	marine	lipids	Human nutrition		[125]
*Phaeodactylum tricornutum*	Phaeophyta	marine	lipids	Human nutrition, biofuels	cDNA libraries, Trans gene expression, *in silico* models	[125,135,136]
*Cyanidioschyzon merolae*	Rhodophyta	freshwater	lipids	Human nutrition, biofuels	Trans gene expression	[137,138]
*Pyropia yezoensis*	Rhodophyta	marine	carotenoids, vitamin B12, PGP glycoprotein, Usujilene—kind of mycosporine-glycine like amino acid	food applications (nori in suschi), anti-inflammatory activity, antioxidative activity	Functional genes cloned	[2,139,140,141,142]
*Gracilariopsis lemaneiformis*	Rhodophyta	marine	fatty acid (12S-hydroxyeicopentaenoic acid-12S-HEPE)	Human nutrition	Protoplast fusion	[143]
*Chondrus crispus*	Rhodophyta	marine	carrageenan	Food applications	Established qPCR references	[96]
*Synechococcus elongatus*	Cyanophyta	freshwater	exopolymers	Carbon cycling, materials applications	Trans gene expression, Tn5 mutagenesis, fusion PCR, CRISPR	[144]
*Anabaena variabilis*	Cyanophyta	freshwater	β-Carotene hydroxylase		Trans gene expression	[145,146,147]
*Anabaena cylindric*	Cyanophyta	freshwater	scytophycin	antifungal activity		[148,149]

^1^ References either for bioactive compounds or genetic tools from listed species in that row only.
